# Quorum sensing and QsvR tightly control the transcription of *vpa0607* encoding an active RNase II-type protein in *Vibrio parahaemolyticus*

**DOI:** 10.3389/fmicb.2023.1123524

**Published:** 2023-01-19

**Authors:** Yiquan Zhang, Xingfan Xue, Fengjun Sun, Xue Li, Miaomiao Zhang, Qimin Wu, Tingting Zhang, Xi Luo, Renfei Lu

**Affiliations:** ^1^Department of Clinical Laboratory, Affiliated Nantong Hospital 3 of Nantong University, Nantong, Jiangsu, China; ^2^School of Medicine, Jiangsu University, Zhenjiang, Jiangsu, China; ^3^Department of Pharmacy, The First Affiliated Hospital of Army Medical University, Chongqing, China

**Keywords:** *Vibrio parahaemolyticus*, quorum sensing, QsvR, VPA0607, RNase II

## Abstract

Vibrio parahaemolyticus, a Gram-negative, halophilic bacterium, is a leading cause of acute gastroenteritis in humans. AphA and OpaR are the master quorum sensing (QS) regulators operating at low cell density (LCD) and high cell density (HCD), respectively. QsvR is an AraC-type protein that integrates into the QS system to control gene expression by directly controlling the transcription of *aphA* and *opaR*. However, the regulation of QsvR itself remains unclear to date. In this study, we show that *vpa0607* and *qsvR* are transcribed as an operon, *vpa0607*-*qsvR*. AphA indirectly activates the transcription of *vpa0607* at LCD, whereas OpaR and QsvR directly repress *vpa0607* transcription at HCD, leading to the highest expression levels of *vpa0607* occurs at LCD. Moreover, VPA0607 acts as an active RNase II-type protein in *V. parahaemolyticus* and feedback inhibits the expression of QsvR at the post-transcriptional level. Taken together, this work deepens our understanding of the regulation of QsvR and enriches the integration mechanisms of QsvR with the QS system in *V. parahaemolyticus*.

## Graphical Abstract

Regulatory circuit. The regulatory relationships between AphA, OpaR and QsvR are confirmed in a previous study (indicated by red lines) (Zhang et al., 2019). The letter ‘P’ together with the thin arrow line represents the transcription start site of the *vpa0607-qsvR* operon. 
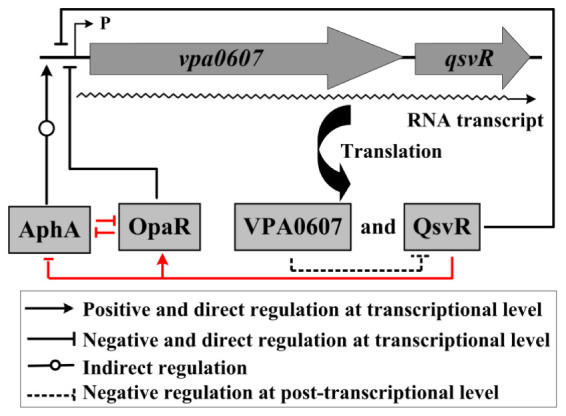

## Introduction

*Vibrio parahaemolyticus* is a Gram-negative, halophilic bacterium that often causes acute gastroenteritis in humans *via* the consumption of seafood (Chen et al., 2022a). In rare cases, *V. parahaemolyticus* is also able to cause wound infection or septicemia if it comes in contact with small open wounds or enters the blood stream (Broberg et al., 2011). Pathogenicity of *V. parahaemolyticus* depends on the production of multiple virulence factors, including thermostable direct hemolysin (TDH), TDH-related hemolysin (TRH), type III secretion systems (T3SS1 and T3SS2) and type VI secretion systems (T6SS1 and T6SS2) (Li et al., 2019). In addition, *V. parahaemolyticus* has a strong capacity to form steady biofilms on the surface, which requires the involvement of structures such as exopolysaccharide (EPS), type IV pili, and flagella, and is also a tightly regulated process (Ashrafudoulla et al., 2021; Liu et al., 2021; Ruhal and Kataria, 2021).

Quorum sensing (QS) is a sophisticated cell density-dependent communication process that involves regulating gene expression and bacterial behaviors through detection of changes in concentration of signal molecules termed autoinducers in growth environments (Lu et al., 2018). QS is known to be involved in controlling motility, biofilm development as well as virulence gene expression (Antunes et al., 2010; Rutherford et al., 2011; Lu et al., 2018; Ruhal and Kataria, 2021; Cai et al., 2022). Interference of QS function could be an alternative therapeutic approach to fight bacterial infection in clinical (Shukla et al., 2021; Rather et al., 2022). In vibrio species, QS employs the master regulators, AphA and OpaR orthologs, to regulate gene expression (Rutherford et al., 2011; Sun et al., 2012; Zhang et al., 2012; Lu et al., 2018). AphA exerts its regulatory roles at low cell density (LCD) to activate biofilm formation, motility, cyclic di-GMP synthesis, and virulence factor production, whereas OpaR or its orthologs operates at high cell density (HCD) to repress these behaviors (Rutherford et al., 2011; Sun et al., 2012, 2022; Zhang et al., 2012, 2021a; Wang et al., 2013a,b; Zhou et al., 2013; Kernell Burke et al., 2015; Lu et al., 2018, 2019, 2021; Chen et al., 2022b). However, OpaR or its orthologs also seems to have regulatory actions at LCD, as it is also expressed under LCD conditions (Rutherford et al., 2011; van Kessel et al., 2013; Wang et al., 2013b; Zhou et al., 2013; Lu et al., 2019). In addition, expression of the master QS regulators seems to be also strictly regulated by numerous regulators in vibrio species (Liu et al., 2006; Tsou et al., 2011; Williams et al., 2012; Zhang et al., 2019, 2023; Cai et al., 2022).

The AraC-family protein QsvR was originally described as a repressor of biofilm formation by *V. parahaemolyticus* (Enos-Berlage et al., 2005). A later study found that QsvR integrated into the QS regulatory cascade by directly regulating the transcription of *aphA* and *opaR* (Zhang et al., 2019). QsvR also operates at HCD to regulate the transcription of a number of genes, including *tdh2*, *toxR*, *calR*, *cpsQ-mfpABC*, *mfpABC* as well as those encoding T3SS1, T3SS2, and T6SS2 (Zhang et al., 2019, 2021b; Qiu et al., 2020). Most importantly, we recently found that QsvR worked coordinately with OpaR to negatively regulate biofilm formation by precisely controlling the transcription of multiple biofilm formation-associated genes including those involved in the biosynthesis of EPS, type IV pili and cyclic di-GMP (unpublished data). However, no potential promoters were detected within the 500 bp upstream of *qsvR* (Zhang et al., 2019), and thus the regulation of QsvR itself remains unknown to date.

The two genes, *vpa0607* and *qsvR*, are transcribed in the same direction on chromosome II and adjacent to each other with an intergenic region of only 71 bp (Makino et al., 2003), suggesting that they may be transcribed as an operon. In addition, an OpaR box-like sequence, AGCTGTTTAATTCATCAATA, was detected within the upstream DNA region of *vpa0607*, indicating that *vpa0607* transcription is likely to be directly regulated by OpaR. The *vpa0607* gene encodes a putative exoribonuclease II, but whether the VPA0607 protein possesses the enzyme activity is also still unknown.

In this study, we demonstrate that *vpa0607* and *qsvR* constitute an operon, *vpa0607*-*qsvR*, and are transcribed as a single primary RNA. AphA indirectly activates the transcription of *vpa0607* at LCD, whereas OpaR and QsvR directly repress *vpa0607* transcription at HCD, leading to the highest expression levels of *vpa0607* occurs at LCD. Moreover, VPA0607 works as an active RNase II-type enzyme in *V. parahaemolyticus* and feedback inhibits the expression of QsvR at the post-transcriptional level. Taken together, this work deepens our understanding of the regulation of QsvR and enriches the integration mechanisms of QsvR with the QS system in *V. parahaemolyticus*.

## Materials and methods

### Bacterial strains

*Vibrio parahaemolyticus* RIMD2210633, which was kindly provided by Dr. Dongsheng Zhou from Beijing Institute of Microbiology and Epidemiology, was used as the wild-type (WT) strain in this study (Makino et al., 2003). The *aphA*, *opaR* and *qsvR* single-gene mutants (Δ*aphA*, Δ*opaR*, and Δ*qsvR*) were constructed by our previous studies (Sun et al., 2012; Zhang et al., 2012, 2019). Δ*aphA*, Δ*opaR*, and Δ*qsvR* have been demonstrated to be non-polar (Sun et al., 2012, 2022; Zhang et al., 2012, 2019, 2021a,2021b; Wang et al., 2013a; Zhou et al., 2013; Qiu et al., 2020; Lu et al., 2021; Chen et al., 2022b), and thus their complementary strains were not included in this study. For over-expression of VPA0607 (Chen et al., 2022b), a polymerase chain reaction (PCR)-generated DNA fragment comprising the coding region of *vpa0607* together with an upstream synthetic Shine-Dalgarno sequence (AGGAGG) was cloned between the *Sma*I and *Sal*I sites of pBAD33containing an arabinose pBAD promoter and a chloramphenicol resistance gene. The recombinant pBAD33 plasmid (pBAD33-*vpa0607*) was introduced into the WT strain to yield the WT/pBAD33-*vpa0607* strain for over-expression of VPA0607. The empty pBAD33 vector was transferred into the WT strain to yield WT/pBAD33 for using as a control strain.

### Growth conditions

*Vibrio parahaemolyticus* was grown as previously described (Zhang et al., 2019). Briefly, overnight cell cultures in 2.5% (w/v) Bacto Heart Infusion (HI) broth (BD Biosciences, USA) were diluted 50-fold into 15 ml of fresh HI broth and then grown at 37°C with shaking at 200 rpm to reach an optical density at 600 nm (OD_600_) value of 1.4. The resulting bacterial cultures were diluted 1000-fold into 15 ml of HI broth for a third round of incubation and then harvested at the required cell densities. When necessary, the medium was supplemented with 50 μg/mL gentamicin, 5 μg/mL chloramphenicol, or 0.1% (w/v) arabinose.

### RNA isolation and quantitative PCR (qPCR)

Total bacterial RNA was extracted using TRIzol Reagent (Invitrogen, USA). RNA quality was detected by agarose gel electrophoresis, and RNA quantity was determined by spectrophotometry. cDNA was generated from 1 μg of total RNA sample using a FastKing First Strand cDNA Synthesis Kit (Tiangen Biotech, China) according to the manufacturer’s instructions. qPCR assays were performed using the Light Cycler system (Roche, Switzerland) together with FastKing One Step RT-qPCR Kit (SYBR) (TIANGEN, China) (Gao et al., 2011; Zhang et al., 2019). Relative mRNA levels of *vpa0607* were determined based on the standard curve of 16S rRNA expression for each RNA preparation. All primers used in this study are listed in Table 1.

**TABLE 1:** Oligonucleotide primers used in this study.

Target	Primers (forward/reverse, 5′-3′)
**Construction of over-expressed strain**	
*vpa0607*	AGCGGAGCTCAGGAGGAATTCACCATGTTTCAAGATAACCCGCTA /AGCGAAGCTTTTATTCTTCACTTACAGTTTGTTC
**Protein expression**	
*vpa0607*	AGCGGGATCCATGTTTCAAGATAACCCGCTA/AGCGAAGCTTTTATTCTTCACTTACAGTTTGTTC
**RT-PCR**	
*vpa0607*	TATGCTAAAAGCGGTGAT/TGGCTGGTGGACGACTAATG
	ATGCTAAAAGCGGTGATTC/AGCCATTCTCGCCAGGTATG
	TGGGTGACACATTGGAAATCG/TGGCTGGTGGACGACTAATG
**qPCR**	
*vpa0607*	GGAAGTGGACAGCAAGAC/AAGCGAGTAAGAGATTGTTC
*qsvR*	TACACCGCCACCCATAACG/AGCCATTCTCGCCAGGTATG
**Primer extension**	
*vpa0607*	/TTGGATTTGCTGCTTAAGTTGG
**LacZ fusion**	
*vpa0607*	GCGCGTCGACACCCGTCTGTGCTTTACCC/GCGCGAATTCTTGGATTTGCTGCTTAAGTTGG
**EMSA**	
*vpa0607*	ACCCGTCTGTGCTTTACCC/TTGGATTTGCTGCTTAAGTTGG
**DNase I footprinting**	
*vpa0607*	GGGACGACTAAGGGAGGC/TTGGATTTGCTGCTTAAGTTGG

### Reverse transcription (RT)-PCR

Total RNA was extracted from the WT strain using TRIzol Reagent (Invitrogen, USA), and then the contaminated DNA was removed using an Ambion DNA-free kit according to the manufacturer’s instructions. RT-PCR was performed similarly as the previously study (Zhang et al., 2018). Briefly, cDNAs were generated using 8 μg of total RNA and 3 μg of random hexamer primers in a 40 μl reaction mixture. A volume of 50 μl of PCR mixture contained 25 μl 2 × Taq PCR Mix (TIANGEN, China), 2 μl of cDNA sample, and 23 μl of nuclease-free water. The parameters for amplification were as follows: 95°C for 5 min; 30 cycles of 94°C for 30 s, 54°C for 50 s, and 72°C for 50 min; and a final extension step of 72°C for 5 min. The PCR products were detected by 1% agarose gel electrophoresis with ethidium bromide staining.

### LacZ fusion and β-galactosidase assay

LacZ fusion assays were performed as previously described (Zhang et al., 2021b). Briefly, the regulatory DNA region of *vpa0607* was cloned into the pHRP309 vector containing a promoter-less *lacZ* gene and a gentamicin resistance gene (Parales and Harwood, 1993). Thereafter, the recombinant pHRP309 plasmid was transferred into WT and the mutant strains. The resulting transformants were cultured and then lysed to measure the β-galactosidase activity in the cellular extracts using a β-Galactosidase Enzyme Assay System (Promega, USA) according to the manufacturer’s instructions. The β-galactosidase activity (represented by the Miller Units) was calculated using the formula: 10^6^ × [(OD_420_−1.75 × OD_550_)/(T × V × OD_600_)] (Zhang et al., 2021b). T and V represent the reaction time (min) and volume (μL), respectively.

### Primer extension

Primer extension assay was similarly performed as previously described (Zhang et al., 2021a,b). Briefly, 10 μg of total RNA were annealed with 1 pmol of 5′-^32^P-labeled anti-sense primer to generate cDNA using a Primer Extension System (Promega, USA) according to the manufacturer’s instructions. The same 5′-^32^P-labeled primer was also used for DNA sequencing with an AccuPower and Top DNA Sequencing Kit (Bioneer, Republic of Korea) according to the manufacturer’s instructions. The products of primer extension and DNA sequencing were analyzed by 8 M urea-6% polyacrylamide gel electrophoresis, and then detected by autoradiography using Fuji Medical X-ray film (Fujifilm, Japan).

### Preparation of 6 × His-tagged proteins

The entire coding regions of *aphA*, *opaR*, *qsvR*, and *vpa0607* were individually cloned into the pET28a vector (Novagen, USA). Each recombinant plasmid encoding the His-tagged protein was transferred into *E. coli* BL21λDE3 for protein expression. Expression and purification of His-AphA, His-QsvR, His-OpaR, and His-VPA0607 were performed as previously described (Sun et al., 2012; Zhang et al., 2012, 2019). The purity of His-tagged proteins was confirmed by sodium dodecyl sulfate-polyacrylamide gel electrophoresis (SDS-PAGE). Purified His-tagged proteins were stored at −80°C.

### Antibody preparation and western blot analysis

Specific polyclonal IgG against His-QsvR from rabbit serum was prepared in the previously study (Zhang et al., 2019). For the western blot analysis (Fang et al., 2014), cleared whole-cell lysate was prepared from harvested bacterial cells by sonication, followed by determination of protein concentrations using a Bio-Rad Protein Assay Kit (Bio-rad, USA). Equal amounts of protein from samples were separated by SDS-PAGE, immunoblotted onto polyvinylidene fluoride membranes (Immobilon P; Millipore, USA), and incubated with primary antibody, then goat anti-rabbit IRDye^®^ 800CW secondary antibody. Signals were detected using an Odyssey Sa Infrared Imaging System (Odyssey Sa, Japan).

### Electrophoretic mobility-shift assay (EMSA)

For the EMSA (Zhang et al., 2012; Kernell Burke et al., 2015), the 5′-ends of the regulatory DNA fragments of *vpa0607* were labeled with [γ-^32^P] ATP. EMSA binding was performed in a 10 μl reaction volume containing 1 mM MgCl_2_, 0.5 mM EDTA, 0.5 mM DTT, 50 mM NaCl, 10 mM Tris–HCl/pH 7.5, 0.05 mg/ml salmon sperm DNA, 5′-^32^P-labeled DNA probe (1000–2000 CPM/μl), and proper amount of His-tagged protein. Two controls were included in each EMSA experiment: (1) cold probe as specific DNA competitor (the unlabeled regulatory DNA fragments of *vpa0607*), and (2) negative probe as non-specific DNA competitor (the unlabeled 16S rRNA gene). Binding products were analyzed in a native 4% (w/v) polyacrylamide gel, and then detected by autoradiography after exposure to Fuji Medical X-ray film (Fujifilm, Japan).

### DNase I footprinting

For DNase I footprinting (Zhang et al., 2012), the regulatory DNA fragments with a single ^32^P-labeled end were generated by PCR, and then purified by a QiaQuick column (Qiagen, Germany). DNA binding was performed in a 10 μl reaction volume, which was the same as the EMSA, and then incubated at room temperature for 30 min. Prior to digestion, 10 μl of Ca^2+^/Mg^2+^ solution (5 mM CaCl_2_ and 10 mM MgCl_2_) were added to each reaction and incubated for 1 min at room temperature. Optimized RQ1 RNase-Free DNase I (Promega, USA) was added to each reaction and then incubated at room temperature for 40–90 s. The reaction was quenched by adding 9 μl of stop solution (200 mM NaCl, 30 mM EDTA, and 1% SDS), followed by incubation for 1 min at room temperature. The partially digested DNA samples were extracted with phenol/chloroform, precipitated with ethanol, and analyzed on a 6% polyacrylamide/8 M urea gel. Protected regions were identified by comparison with the DNA sequence ladders. Radioactive species were detected by autoradiography after exposure to Fuji Medical X-ray film (Fujifilm, Japan).

### Enzyme activity assay

The RNase II activity of His-VPA0607 was performed similarly as previously described (Schmier et al., 2012). Briefly, the substrate consisting of a 17-base pair duplex with a 17-nucleotide 3′ poly (A) overhang (ds17-A_17_) was prepared by mixing the oligoribonucleotide 5′- CCCCACCACCAUCACUUAAAAAAAAAAAAAAAAA-3′ with the complementary oligoribonucleotide 5′-AAGUGAUGGUGGUGGGG-3′ in a 1:1.2 molar ratio in the presence of 10 mM Tris–HCl (pH 8.0) and 20 mM KCl, heating the mixture in a boiling water bath for 5 min, and then allowing the solution to cool slowly at room temperature. RNase II activity of His-VPA0607 was performed in 50 μl reaction mixtures containing 50 mM Tris–HCl (pH 8.0), 100 mM KCl, 10 mM MgCl_2_, 5 mM DTT, 2 μM of substrate, and 48 μg of purified His-VPA0607. Reaction mixtures were incubated at 37°C, with 4 μl aliquots removed at the indicated time points and terminated with 2 volumes gel loading buffer containing 95% formamide, 20 mM EDTA, 0.025% bromophenol blue, and 0.025% xylene cyanol. Reaction products were analyzed by a native 12% (w/v) polyacrylamide gel, and the gel images were displayed with an ultraviolet (UV) transilluminator.

### Experimental replicates and statistical methods

The qPCR and LacZ fusion assays were performed at least three times, with the values were expressed as the mean ± standard deviation (SD). A paired Student’s *t*-test was used to calculate statistically differences with *P* < 0.01 considered as significant. The primer extension, western blot, EMSA, DNase I footprinting and enzyme activity assays were performed at least three independent times.

## Results

### *vpa0607* and *qsvR* constitute an operon

**FIGURE 1:**
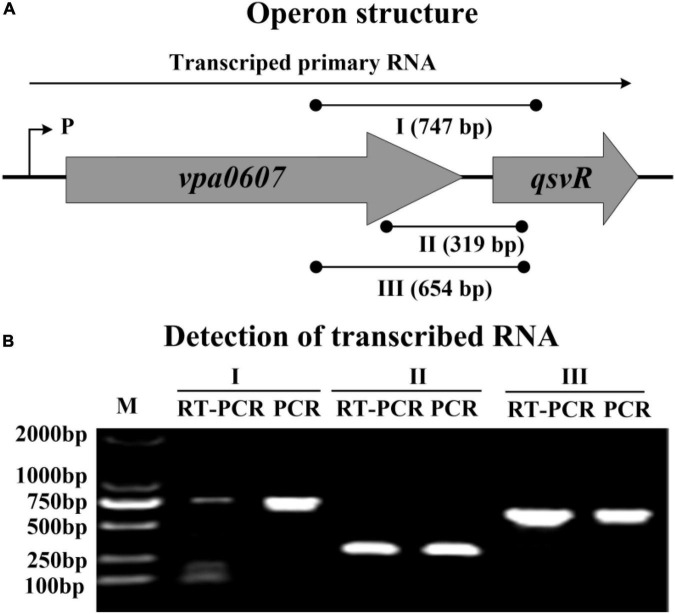
Transcriptional organization of the *vpa0607*-*qsvR* operon. **(A)** Operon structure. The boxed arrows represent the coding regions of corresponding genes. The broken arrow with a “P” alongside indicates the transcription start site. The long horizontal arrow depicts the putative primary RNA transcript. The lines with black dots indicate the location of primer pairs and the theoretical amplicons of PCR. I, II, and III represent different primer pairs. Numbers in parentheses indicate the theoretical length of amplicons. **(B)** Detection of transcribed RNA. cDNA samples were generated by RT from the total RNAs of WT. Genomic DNA and cDNA were used as the templates for PCR and RT-PCR, respectively. Two blank controls were included: (1) PCR reactions without template; (2) PCR reactions with the total RNA only treated by DNase I as the template. As expected, both blank controls did not provide any amplicons (data not shown).

We noticed that *vpa0607* and *qsvR* are placed adjacent to each other on the chromosome II, and transcribed in the same direction with a length of 71 bp intergenic DNA region (Makino et al., 2003). Therefore, we performed RT-PCR to test if *vpa0607* and *qsvR* are co-transcribed. As shown in Figure 1, these two genes were demonstrated to constitute an operon, *vpa0607*-*qsvR*, transcribed as a single primary RNA. *vpa0607* is the first gene of the *vpa0607*-*qsvR* operon.

### Cell-density dependent transcription of *vpa0607*

**FIGURE 2:**
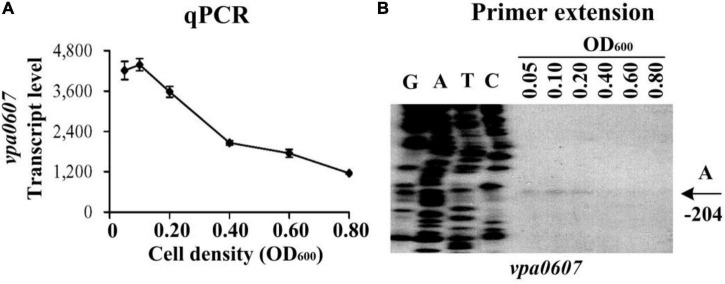
Cell density-dependent transcription of *vpa0607*. The wild-type (WT) strain was grown in HI broth at 37°C with shaking at 200 rpm, and then harvested at different cell densities. **(A)** qPCR. Relative mRNA levels of *vpa0607* were examined in the WT strain at different cell densities. **(B)** Primer extention. The products of primer extension were detected by an 8 M urea-6% acrylamide sequencing gel. Lanes G, A, T, and C represent Sanger sequencing reactions. Transcription start site of *vpa0607* is indicated by arrows with nucleotide and position. The minus number indicates the nucleotide position upstream of the first base of the start codon of *vpa0607*.

Expression of QsvR manifested a cell-density dependent manner, and a high expression level was observed for QsvR at HCD (Zhang et al., 2019). The fact that *vpa0607* is co-transcribed with *qsvR* promoted us to detect the expression changes of *vpa0607* in the WT strain across growth periods. The results of qPCR showed that the mRNA levels of *vpa0607* decreased considerably with the increase of cell density from an OD_600_ value of 0.05 to 0.8 (Figure 2A). As further determined by primer extension (Figure 2B), the *vpa0607* mRNA was only capable of being detected at an OD_600_ value of 0.05 to 0.2; when the OD_600_ value was higher than 0.2, the *vpa0607* mRNA was undetectable; in addition, the nucleotide A, locating at 204 bp upstream of the first base of the start codon (+ 1), was thought to be the transcription start site of *vpa0607*. In brief, transcription of *vpa0607* was cell-density dependent, and the highest transcription levels occurred at LCD.

### Regulation of *vpa0607* by the master QS regulators

The cell density-dependent transcription of *vpa0607* encouraged us to detect whether the transcription of *vpa0607* was regulated by the master QS regulator, AphA, and OpaR. Indeed, an OpaR box-like sequence, AGCTGTTTAATTCATCAATA, was detected in the promoter DNA region of *vpa0607*-*0606*. Therefore, the bacterial cells were harvested at an OD_600_ value of approximately 0.15 and 0.8 to simulate LCD and HCD conditions (Sun et al., 2012; Zhang et al., 2012, 2019), respectively, to investigate AphA- and OpaR-mediated *vpa0607* transcription.

**FIGURE 3:**
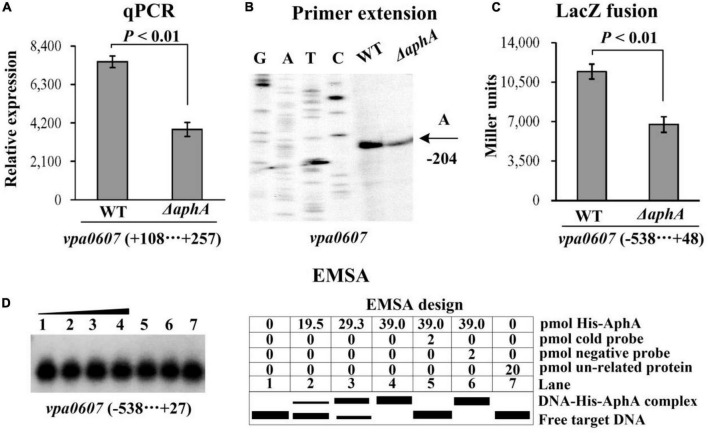
Regulation of *vpa0607* transcription by AphA. Bacterial cells were harvested at OD_600_ value of approximately 0.15 to investigate AphA-mediated *vpa0607* transcription. Negative and positive numbers indicate the nucleotide positions upstream and downstream of *vpa0607*, respectively. **(A)** qPCR. Relative mRNA levels of *vpa0607* were compared between Δ*vpa0607* and WT. Primer extension **(B)** was performed as described in Figure 2. **(C)** LacZ fusion. The regulatory DNA region of *vpa0607* was cloned into the pHRP309 vector and then, respectively, transferred into Δ*aphA* and WT to determine the promoter activity (Miller units) in the cellular extracts. **(D)** EMSA. The radioactively labeled regulatory DNA fragments of *vpa0607* were incubated with increasing amounts of purified His-AphA and then subjected to 4% (w/v) polyacrylamide gel electrophoresis. Shown on the right is the EMSA design.

**FIGURE 4:**
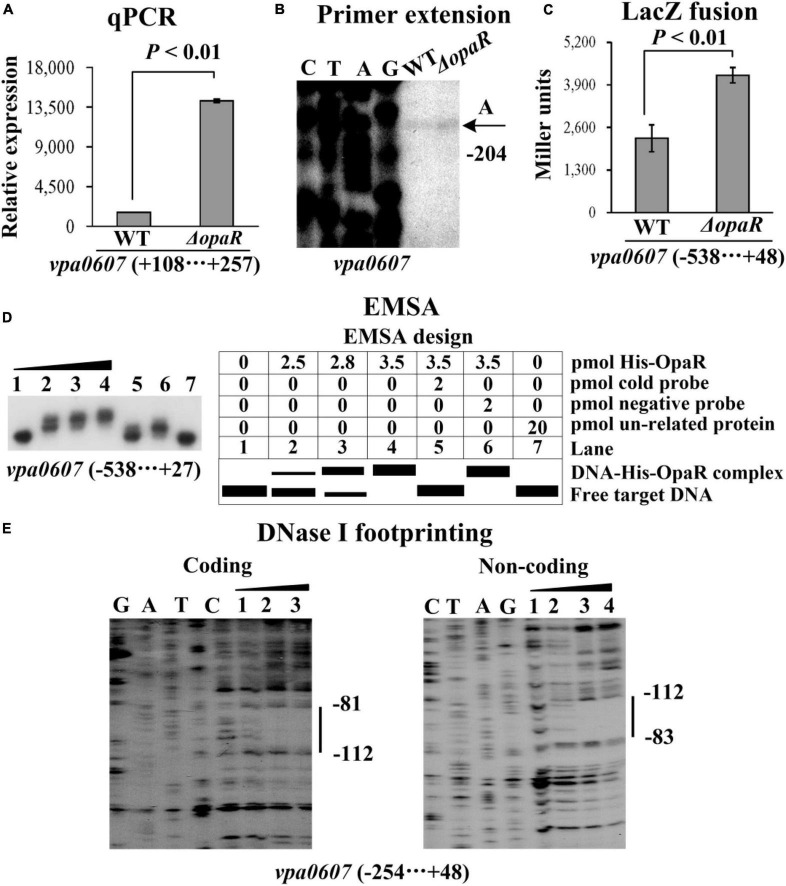
Regulation of *vpa0607* transcription by OpaR. Bacterial cells were harvested at OD_600_ value of approximately 0.8 to investigate OpaR-mediated *vpa0607* transcription. Negative and positive numbers indicate the nucleotide positions upstream and downstream of *vpa0607*, respectively. Primer extension **(B)** was performed as described in Figure 2. qPCR **(A)**, LacZ fusion **(C)**, and EMSA **(D)** were performed as described in Figure 3. **(E)** DNase I footprinting. Labeled coding or non-coding DNA probes were incubated with increasing amounts of purified His-OpaR and then subjected to DNase I footprinting. The protected regions are indicated by vertical bars, with the corresponding sequence positions also indicated. Lanes C, T, A, and G represent the Sanger sequencing reactions.

As determined by qPCR (Figures 3A, 4A), the mRNA levels of *vpa0607* were significantly decreased in Δ*aphA* but increased in Δ*opaR* relative to those in WT. The results of primer extension assay further indicated that the mRNA levels of *vpa0607* were decreased in Δ*aphA* while increased in Δ*opaR* relative to those in WT (Figures 3B, 4B). The recombinant *lacZ* fusion pHRP309 plasmid containing the promoter DNA region of *vpa0607* and a promoterless *lacZ* gene was transformed into Δ*aphA*, Δ*opaR*, and WT, respectively, to investigate whether AphA and OpaR have regulatory actions on the promoter activity of *vpa0607*. As shown in Figures 3C, 4C, the promoter activity of *vpa0607* was significantly decreased in Δ*aphA* whereas enhanced in Δ*opaR* relative to that in the WT strain. The results of *in vitro* EMSA showed that His-AphA did not bind to the upstream DNA fragment of *vpa0607*, while His-OpaR was able to specially and dose-dependently bind to this DNA fragment (Figures 3D, 4D). However, His-AphA was capable of binding to other promoters such as its own promoter at much lower amount of protein (Sun et al., 2012). As further determined by the DNase I footprinting assay (Figure 4E), His-OpaR protected a single DNA region within the upstream DNA region of *vpa0607*, located 112 to 81 bp upstream of the translation start site, against DNase I digestion. Taken together, the transcription of *vpa0607* was activated indirectly by AphA at LCD, but was repressed directly by OpaR at HCD.

### QsvR represses *vpa0607* transcription

**FIGURE 5:**
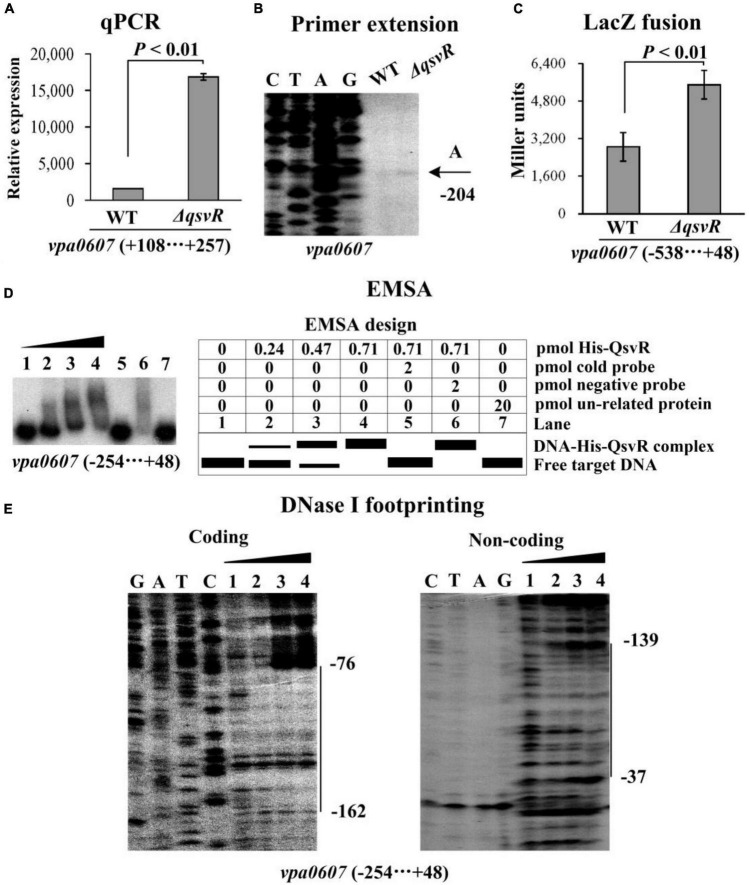
Regulation of *vpa0607* transcription by QsvR. Bacterial cells were harvested at OD_600_ value of approximately 0.8 to investigate QsvR-mediated *vpa0607* transcription. Negative and positive numbers indicate the nucleotide positions upstream and downstream of *vpa0607*, respectively. Primer extension **(B)** was performed as described in Figure 2. qPCR **(A)**, LacZ fusion **(C),** and EMSA **(D)** were performed as described in Figure 3. DNase I footprinting **(E)** was performed as described in Figure 4.

QsvR integrates into the QS cascade to control gene expression in *V. parahaemolyticus*, and is highly expressed at HCD (Zhang et al., 2019). In this study, the bacterial cells were harvested at an OD_600_ value of approximately 0.8 to investigate whether QsvR has regulatory activity on the transcription of *vpa0607* (Zhang et al., 2019). The results of qPCR and primer extension assays showed that the mRNA level of *vpa0607* was significantly enhanced in Δ*qsvR* relative to that in WT (Figures 5A, B). The *lacZ* fusion results demonstrated that the promoter activity of *vpa0607* in Δ*qsvR* was significantly enhanced relative to that in WT (Figure 5C). The EMSA results showed that His-QsvR was able to specially and dose-dependently bind to the upstream DNA fragment of *vpa0607* (Figure 5D). As further determined by the DNase I footprinting assay (Figure 5E), His-QsvR protected only one DNA region upstream of *vpa0607*, located 162 to 37 bp upstream of the translation start site, against DNase I digestion, which was considered as the QsvR site. Taken together, QsvR directly repressed the transcription of *vpa0607* at HCD.

### VPA0607 acts as an active RNase II

The *vpa0607* and *qsvR* genes are transcribed as a single primary mRNA (Figure 1). However, the *vpa0607* mRNA was only detected at LCD (Figure 2), whereas QsvR was highly expressed at HCD (Zhang et al., 2019). This pt paradoxical phenomenon prompted us to look deeper into the possible mechanisms behind it. The function of VPA0607 was annotated as an exoribonuclease II, which is involved in hydrolyzing single-stranded mRNA processively in the 3′ to 5′ direction (Makino et al., 2003). We compared the amino acid sequence of VPA0607 with that of *E. coli* RNase II and found that they shared more than 50% identity in amino acid sequence, especially, the critical residue, Y253 (Barbas et al., 2008), at the active site was conserved between the two proteins (Supplementary Figure 1), suggesting that VPA0607 might possess a similar structure and functions with the *E. coli* RNase II. Therefore, we deduced that VPA0607 may function as an active RNase II to regulate QsvR expression at the post-transcriptional level.

**FIGURE 6:**
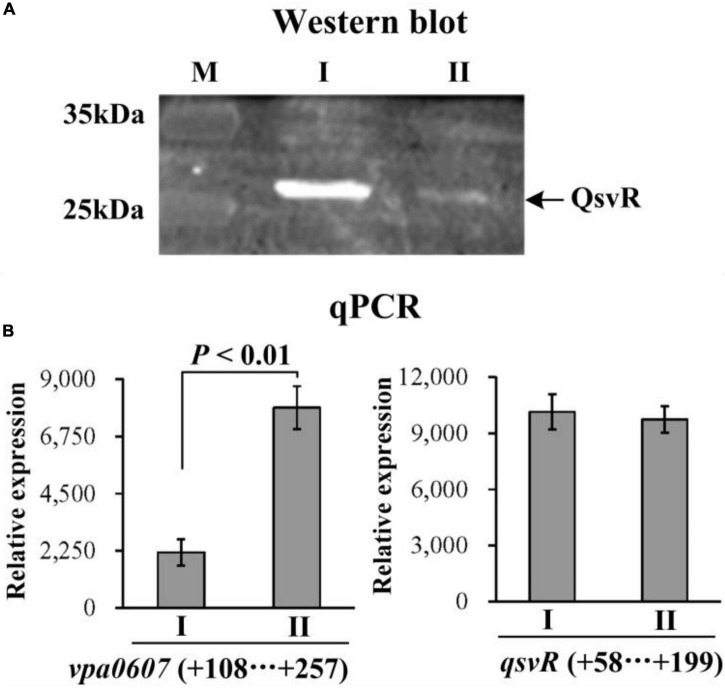
Post-transcriptional regulation of *qsvR* by VPA0607. The WT/pBAD33-*vpa0607* and WT/pBAD33 strains were grown in HI broth containing 5 μg/mL chloramphenicol and 0.1% (w/v) arabinose, and then harvested at an OD_600_ value of approximately 0.8. I and II indicate WT/pBAD33 and WT/pBAD33-*vpa0607*, respectively. **(A)** Western blot. Whole-cell proteins extracted from WT/pBAD33-*vpa0607* and WT/pBAD33 were subjected to SDS-PAGE and then incubated with anti-QsvR antibodies. Proteins were then detected using an Odyssey Sa Infrared Imaging System. **(B)** qPCR was performed as described in Figure 3.

We first performed western blot and qPCR to test whether over-expression of VPA0607 regulates QsvR expression. The results of western blot assays showed that the QsvR level was significantly reduced in the WT/pBAD33-*vpa0607* strain relative to that in the WT/pBAD33 strain (Figure 6A). As further determind by the qPCR assays (Figure 6B), the mRNA level of *vpa0607* in the WT/pBAD33-*vpa0607* strain was significantly higher than that in the WT/pBAD33 strain, while no significant difference was observed for the *qsvR* mRNA levels between the two strains. These results suggested that over-expression of VPA0607 negatively regulated the expression of QsvR.

**FIGURE 7:**
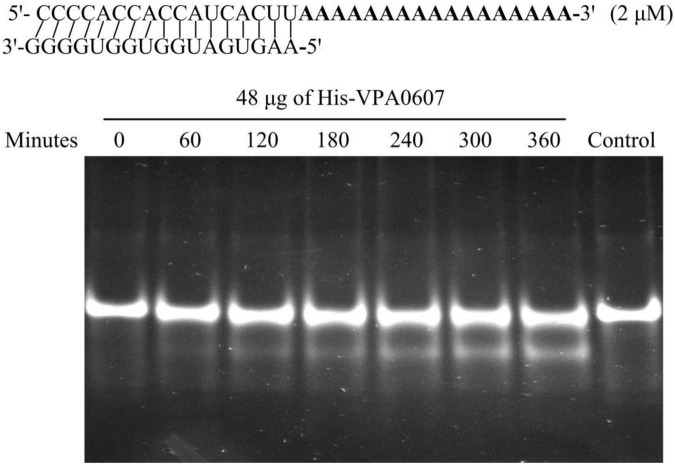
VPA0607 was an RNase II type enzyme. A total of 50 μl reactions were performed with the indicated substrate (a duplex with a 17 nt overhang) and purified His-VPA0607. A total of 4 μl aliquots were removed at the specified time points and analyzed by a native 12% (w/v) polyacrylamide gel, and the gel images were displayed with an ultraviolet (UV) transilluminator. The control was a reaction without His-VPA0607 that was incubated at 37°C for 360 min.

RNase II is a single-stranded, sequence-independent, 3′ exoribonuclease with high hydrolytic activity toward poly (A) RNA (Andrade et al., 2009). To test whether VPA0607 is an RNase II-type enzyme, purified His-VPA0607 was incubated with the ds17-A_17_ in a 50 μl enzymatic reaction system (Schmier et al., 2012). As shown in Figure 7, smaller fragments were detected below the main bands and the smaller bands became brighter and brighter with the extension of incubation time. In contrast, no small bands were detected in the initial and control reactions. These results indicated that VPA0607 was able to digest the 17 nt ssRNA overhang of the substrate, and VPA0607 was likely to be an RNase II-type enzyme in *V. parahaemolyticus*.

## Discussion

**FIGURE 8:**
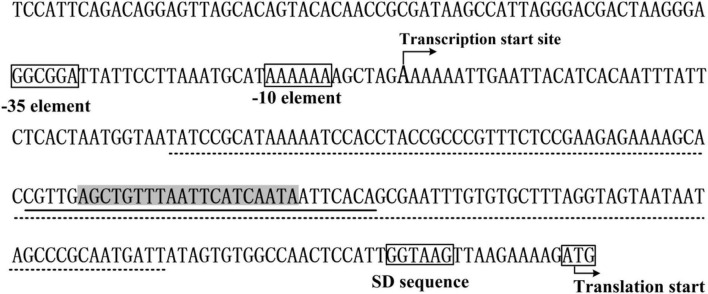
Promoter organization of *vpa0607*. The promoter-proximal DNA region of *vpa0607* were derived from the *Vibrio parahaemolyticus* strain RIMD221063. The translation and transcription start sites are indicated with bent arrows. The predicted –10 and –35 elements as well as the Shine-Dalgarno (SD) sequence are boxed. The OpaR box-like sequence is highlighted in gray. The OpaR site is underlined with solid line, while the QsvR site is underlined with dotted line.

The data presented here shows that *vpa0607* and *qsvR* constitute an operon, *vpa0607*-*qsvR* (Figure 1). The highest transcriptional levels of *vpa0607* occurs at OD_600_ values of 0.2 to 0.4 (Figure 2), suggesting that the transcription of *vpa0607*-*qsvR* is probably regulated by QS. Indeed, the data shows that the transcription of *vpa0607*-*qsvR* is activated indirectly by AphA at LCD, but is repressed directly by OpaR at HCD (Figures 3, 4). Moreover, QsvR is able to bind to the regulatory DNA region of its own operon to repress its transcription (Figure 5). Both the OpaR and QsvR sites for *vpa0607*-*qsvR* are located downstream of the transcription start site (Figure 8). The binding of OpaR or QsvR may block the elongation of the RNA polymerase, and thus, to repress the transcription of *vpa0607*-*qsvR*. Although the QsvR site entirely overlaps the OpaR site, there is very likely to be no competitive binding between the two regulators for the regulatory DNA region of *vpa0607*-*qsvR* according to our unpublished data. Collectively, these data demonstrate that QsvR coordinates with QS regulators, AphA, and OpaR, to precisely regulate the transcription of *vpa0607*-*qsvR* in *V. parahaemolyticus*, leading to the highest transcriptional levels of this operon occurs at LCD.

Some data in this study are incomprehensible to us, that is, QsvR is highly expressed at HCD (Zhang et al., 2019), while *vpa0607* is highly transcribed at LCD, but they are co-transcribed. VPA0607 shares high identity in amino acid sequence with *E. coli* RNase II (Supplementary Figure 1), which is an exoribonuclease that processively degrades RNA from the 3′-end (Andrade et al., 2009). Over-expressed VPA0607 represses QsvR expression at the post-transcriptional level (Figure 6), and the purified VPA0607 protein is able to degrade the ds17-A_17_ *in vitro* (Figure 7). The enzyme activity of RNase II is sequence-independent, but it prefers to degrade substrates with poly(A) tails (Cheng and Deutscher, 2002). Thus, VPA0607 probably acts as an RNase II-type enzyme in *V. parahaemolyticus*. RNase II is a component of the RNA degradosome, playing a central role in RNA processing and degradation (Lu and Taghbalout, 2014). In *E. coli*, RNase II was shown to be required for cell survival during stationary phase and upon starvation by regulating the amount and stability of RNase PH (Sulthana et al., 2017). The transcriptomic RNA-seq data showed that the deletion of RNase II significantly affected the transcription of 187 genes relative to that of the WT strain, including those involved in flagellum assembly, motility and biofilm formation (Pobre and Arraiano, 2015). Indeed, the RNase II mutant produced more biofilm than the WT strain (Pobre and Arraiano, 2015). The roles of VPA0607 are completely unknown in *V. parahaemolyticus* and worth to be investigated in the future.

Regulation of RNase II has been observed in *E. coli*, but mostly at the post-transcriptional level. For example, inactivation of RNase E and RNase III leads to altered expression levels and activity of RNase II (Zilhao et al., 1995). The Cys284Tyr mutation abolishes RNase II activity by increasing protein kinetic instability at the non-permissive temperature (Reis et al., 2019). The acetyltransferase Pka and the deacetylase CobB can determine whether the Lys501 residue in RNase II is acetylated, and thus affecting the catalytic activity of RNase II (Song et al., 2016). The putative RNase II, VPA0607, was transcriptionally regulated by QsvR and the QS system, but the post-transcriptional regulation of VPA0607, especially whether it can interact with other endonucleases, is still worthy of further in-depth study.

In conclusion, this study shows that *vpa0607* and *qsvR* are co-transcribed as an operon, and the transcription of this operon is tightly regulated by QsvR and the master QS regulators, AphA, and OpaR. In addition, VPA0607 is shown to be an active RNase II-type enzyme in *V. parahaemolyticus* and feedback inhibits QsvR expression at the post-transcriptional level. The data of this study prompts us to deduce a hypothesis as following: at LCD, the *vpa0607*-*qsvR* operon is highly transcribed and thus leading to a high intracellular level of VPA0607, which then negatively regulate QsvR expression at the post-transcriptional level by degrading the mRNA of *vpa0607*-*qsvR* from the 3′ ends; at HCD, the intracellular VPA0607 level is low due to the collective repression of QsvR and OpaR, and thus the post-transcriptional repression of QsvR by VPA0607 is abolished and the basal mRNA level of *vpa0607*-*qsvR* can translate QsvR. However, this hypothesis needs to be further investigated.

## Data availability statement

The original contributions presented in this study are included in the article/Supplementary material, further inquiries can be directed to the corresponding author.

## Author contributions

YZ, XX, FS, XuL, MZ, QW, TZ, and XiL performed the laboratory experiments and analyzed the results. YZ and RL designed, organized, and supervised the experiments. YZ drafted the manuscript. All authors read and approved the final manuscript.

## Supplementary material

Supplementary Figure 1 Comparative homology of VPA0607 and *E. coli* RNase II. The amino acid sequences were derived from the *V. parahaemolyticus* strain RIMD2210633 and *E. coli* K12. Identical residues are marked with asterisks (*). The key amino acid residue (Y) is labeled in red color.
