# Supplementary material for: Quorum sensing and QsvR tightly control the transcription of vpa0607 encoding an active RNase II-type protein in Vibrio parahaemolyticus
Source: Front Microbiol. 2023 Jan 19;14:1123524. doi: 10.3389/fmicb.2023.1123524 (PMC9894610; doi:10.3389/fmicb.2023.1123524)
Supplement: Supplementary Figure 1 — Comparative homology of VPA0607 and E. coli RNase II. The amino acid sequences were derived from the V. parahaemolyticus strain RIMD2210633 and E. coli K12. Identical residues are marked with asterisks (*). The key amino acid residue (Y) is labeled in red color. [file Image_1.pdf]

```
V.parahaemolyticus VPA0607  MFQDNPLLAQLKQQIQENLPKKEGSIKATDKGFGFLEVDSKTSFFIPPAYMKKCIHGDKV   60
E.coli RNase II             MFQDNPLLAQLKQQLHSQTPRAEGVVKATEKGFGFLEVDAQKSYFIPPPQMKKVMHGDRI   60
                            **************     *  **  *** *********   * ****  ***  ***

V.parahaemolyticus VPA0607  VAIIRTENEREVAEPQELIEQSLTRFIGRVKMFKGKLNVVPDHPQLKKLSLKAKLKKGLK   120
E.coli RNase II             IAVIHSEKERESAEPEELVEPFLTRFVGKVQGKNDRLAIVPDHPLLKD-AIPCRAARGLN   120
                             * *  * *** *** ** *  **** * *      *  ***** **          **

V.parahaemolyticus VPA0607  PDNFAEGDWVVAHLVRHPLKGDNTFFVEISEKITDADDKIAPWWVTLAQNDLPNSEPAGI   180
E.coli RNase II             HE-FKEGDWAVAEMRRHPLKGDRSFYAELTQYITFGDDHFVPWWVTLARHNLEKEAPDGV   178
                               * **** **   *******  *  *    **  **   *******   *    * *

V.parahaemolyticus VPA0607  ENWELKDDADLERIEMTHVPFVTIDGESTKDMDDALYAKKTESGDFELTIAIADPTAYIT   240
E.coli RNase II             ATEML--DEGLVREDLTALDFVTIDSASTEDMDDALFAKALPDDKLQLIVAIADPTAWIA   236
                                *  *  * *   *   *****  ** ****** **        *  ******* *

V.parahaemolyticus VPA0607  PEDEMDKVARERGYTIYLPGRNIPMLPRDLADNLCSLIEGEIRPAICCTVTVSKDGVIGD   300
E.coli RNase II             EGSKLDKAAKIRAFTNYLPGFNIPMLPRELSDDLCSLRANEVRPVLACRMTLSADGTIED   296
                                 ** *  *  * **** ******* * * ****   * **   *  * * ** * *

V.parahaemolyticus VPA0607  DIKFFAANIKSHARLAYDHVSDWLENGNSDAWQP-SEEIATIVRDLYEFSLARAEWREKN   359
E.coli RNase II             NIEFFAATIESKAKLVYDQVSDWLENTGD--WQPESEAIAEQVRLLAQICQRRGEWRHNH   354
                             * **** * * * * ** *******     *** ** **  ** *      * ***

V.parahaemolyticus VPA0607  AVVFPDRPDYRFELSEDNDVIAIHADMRRSANRLVEESMITANICAGRTLREKFETGVFN   419
E.coli RNase II             ALVFKDRPDYRFILGEKGEVLDIVAEPRRIANRIVEEAMIAANICAARVLRDKLGFGIYN   414
                            * ** ******* * *   *  * *  ** *** *** ** ***** * ** *   *  *

V.parahaemolyticus VPA0607  THAGLKPEKIEEVVQLVNPEGTLEFTAESIATLEGFAALRRWLAVQETSYLDNRIRKFQA   479
E.coli RNase II             VHMGFDPANADALAALLKTHG-LHVDAEEVLTLDGFCKLRRELDAQPTGFLDSRIRRFQS   473
                             * *  *        *    * *   **   ** **  *** *  * *  ** *** **

V.parahaemolyticus VPA0607  YSEVGNQPLPHYAMGLDIYATWTSPIRKYGDMINHRMLKAVILDKEPVQKPDDQVGEELA   539
E.coli RNase II             FAEISTEPGPHFGLGLEAYATWTSPIRKYGDMINHRLLKAVI-KGETATRPQDEITVQMA   532
                              *      **   **  ****************** *****   *      *      *

V.parahaemolyticus VPA0607  LHRKHHKIAERNVSDWLYARTLADEPSKQTCFTGEIFDINRAGARVRLLENGAAAFIPGA   599
E.coli RNase II             ERRRLNRMAERDVGDWLYARFLKDKAGTDTRFAAEIVDISRGGMRVRLVDNGAIAFIPAP   592
                              *     *** * ****** * *     * *  ** ** * * ****  *** ****

V.parahaemolyticus VPA0607  LILDNKERIECNGDNGTISIDKEVVYKLGDTLEIVLADVNQENRSLVAKPT   650
E.coli RNase II             FLHAVRDELVCSQENGTVQIKGETVYKVTDVIDVTIAEVRMETRSIIARPV   643
                                      *   ***  *  * ***  *      * *  * **  * *
```

Figure S1
